# How individual BMI affected general cognitive ability in young adults: a moderated chain mediation model

**DOI:** 10.3389/fpubh.2025.1559582

**Published:** 2025-03-20

**Authors:** KeZhen Lv, ShengJie Xu, YuQi Sun, Rui Zhou, Hanyuan Xu, Junhao He, Cheng Xu, Hui Xu, Jing Xu, Jun Qian

**Affiliations:** ^1^School of Mental Health, Wenzhou Medical University, Wenzhou, China; ^2^Ministry of Education College Student Mental Health and Comprehensive Quality Training Base, Wenzhou Medical University, Wenzhou, China

**Keywords:** BMI, general cognitive ability, sleep quality, impulsive trait, chain mediation model, gender differences

## Abstract

**Objective:**

With the rising global obesity rates, increasing research has been directed toward understanding how obesity affects cognitive ability in young adults. This study aims to explore the impact of body mass index (BMI) on general cognitive ability and how sleep quality and impulsive trait mediate this relationship.

**Methods:**

A total of 1,205 young adults from Human Connectome Project(HCP) project were included, and questionnaires and cognitive assessment tools were conducted.

**Results:**

BMI was negatively correlated with general cognitive ability, with sleep quality and impulsive trait acting as chain mediators between BMI and general cognitive ability. Additionally, gender moderated the effect of BMI on sleep quality, with this effect being more pronounced in female young adults.

**Conclusion:**

This study not only provided new insights into the impact of BMI on general cognitive ability in young adults but also offered an important perspective on how sleep quality and impulsive trait influenced this process. These findings provide a scientific basis for preventive measures against obesity and cognitive impairment in young adults.

## 1 Introduction

Globally, obesity has become an increasingly severe public health issue. According to data from the World Health Organization in 2021, the number of individuals with obesity worldwide has tripled since 1975 (1). Obesity has been shown to be associated with all-cause mortality and poorer health outcomes, and it may impair cognitive function (2). A survey in 2024 on the global prevalence rate of obesity in 99% of the population revealed that obesity-related symptoms have become predominant in adults and are rapidly obeserved among young individuals, with the double prevalence rate of youth with obesity among most countries (3). This means that obesity among teenagers is gradually becoming a common problem that needs urgent attention. However, current research mostly focuses on the entire age range of individuals with obesity, with few studies specifically examining the characteristics of obesity in large samples of young adults.

Body mass index (BMI) is one of the indicators used to measure the degree of obesity in individuals. High BMI is almost universally associated with declines in cognitive ability across all domains (4, 5). General cognitive ability can be divided into fluid cognitive ability and crystallized cognitive ability. Fluid intelligence primarily involves the ability to process new information, logical reasoning, and solving new problems, while crystallized intelligence involves knowledge and skills accumulated through experience and education (6). Executive function, as a type of fluid intelligence, is often impaired by inflammation factors induced by obesity, affecting working memory and inhibitory control (7). It has found that students with high BMI have significantly lower academic performance compared to those with normal weight, indicating that their crystallized intelligence is also impaired (5). Therefore, we hypothesized that high BMI may damage an individual’s general cognitive ability. Previous research investigated how BMI affects cognition from a physiological perspective (8), while few studies have taken psychological factors as mediators into account. Research indicates that the interaction between physiological and psychological factors plays a crucial role in the onset and development of obesity (9). Furthermore, the relationship between BMI and general cognitive ability inherently involves a transition from physiological to psychological factors, necessitating the interaction of these factors. Therefore, we propose constructing a chain mediation model comprising both physiological and psychological factors to explain the relationship between obesity and cognitive ability.Sleep quality refers to personal overall sleep health and is often influenced by physiological functions such as cardiovascular function (10).

Among these psychological factors, sleep quality and impulsive traits are particularly noteworthy. Sleep quality refers to personal overall sleep health and is often influenced by physiological functions such as cardiovascular function (10). In non-clinical populations, sleep quality is typically assessed using the Pittsburgh Sleep Quality Index (PSQI) (11). Poor sleep quality is often associated with elevated BMI, as individuals with high BMI tend to consume unhealthy foods characterized by high fat and carbohydrate intake and poor diet quality can affect sleep quality (12, 13). Additionally, poor sleep quality can lead to a decline in cognitive ability (14–16). Previous research has found that sleep quality played a crucial mediating role in the relationship betwwen BMI and their cognitive function in children (17), but there is less evidence supporting this mediating relationship in young adults.

Beyond sleep disturbances, individuals with higher BMI also tend to exhibit greater impulsivity, which further contributes to cognitive deficits. Impulsive trait is a tendency to act on desires and urges without considering the consequences and is a cognitive trait that operates in specific situations (18, 19). Individuals with high BMI often exhibit higher levels of impulsivity (20), possibly due to neuroendocrine abnormalities that make it more difficult for them to resist cravings for food (21). Higher BMI is associated with reductions in the volume of various brain regions involved in reward and impulse control, such as the nucleus accumbens and striatum. These changes may lead to diminished control over food intake and other impulsive behaviors (22). The increase in impulsivity is associated with a decline in overall cognitive ability (23). For instance, attentional impulsivity and non-planning impulsivity significantly contribute to the reduction of cognitive abilities (24). This suggests that impulsivity may mediate the relationship between BMI and general cognitive ability, although this has not yet been empirically verified. Delay discounting, which refers to the preference for smaller immediate rewards over larger delayed rewards, is an external manifestation of impulsivity. High levels of delay discounting are associated with weaker self-control, higher impulsivity, and greater immediate gratification needs (25). Higher levels of delay discounting are often associated with greater possibility of becoming obesity, as individuals with obesity tend to have a weaker ability to inhibit their cravings for food (25), thus this study will adopt this experimental method.

Given the strong association between high impulsivity and poor sleep quality, these two variables may not contribute solely to the relationship between BMI and general cognitive ability (26–28). Researchers found that reduced sleep duration and abnormal sleep patterns can make individuals more impulsive and prone to taking risks (29–31). This may be due to the negative impact of decreased sleep quality on function activity of prefrontal cortex, which is responsible for higher-level cognitive processes, including judgment and decision-making (30). In another study, participants underwent 8 days of normal sleep followed by 3 days of sleep deprivation, during which they slept 2 h less each day (31). It has found that sleep deprivation significantly increased impulsivity and decreased the positive affect experienced by individuals. Conversely, good sleep quality can lead to positive emotions, which help enhance self-control abilities (32). Previous study also pointed out that individuals with better sleep quality tended to perform better on delay discounting tasks (33). Given the correlation between sleep quality and impulsivity, we propose that sleep quality and impulsivity will serve as chain mediators in the relationship between BMI and general cognitive ability.

Among the various impacts triggered by BMI, gender often serves as a moderating factor. This is likely due to differences between males and females in body fat distribution, hormone levels, and health risks associated with obesity (34). The pattern of BMI’s influence on general cognitive ability varies by gender (35). Across all age groups, BMI has a greater effect on general cognitive ability in male than that in female (36). Furthemore, women’s sleep quality, rather than men’s, is more likely to deteriorate with increasing BMI (34, 37). High BMI is significantly associated with both excessively long and short sleep durations in women, whereas it is only associated with excessively long sleep durations in men (34). Based on these findings, we hypothesize that gender may moderate the impact of BMI on sleep quality, leading to changes in general cognitive ability.

**Figure 1:**
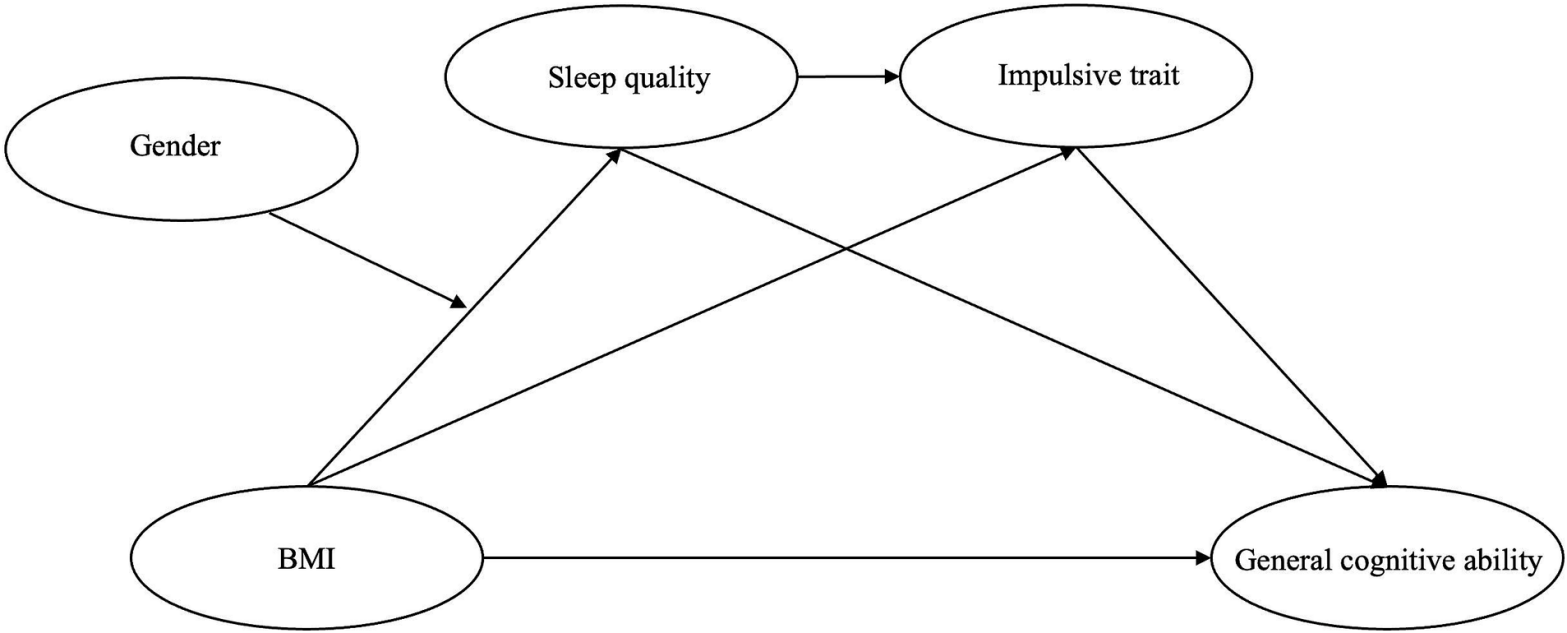
A proposed model in this study. BMI, body mass index.

Considering that a single mediation model can usually only reveal the role of one mediating variable between the independent variable and the dependent variable, the chain mediation model provides a more detailed perspective, which can reveal how multiple mediating variables work together and influence each other, thereby providing a deeper understanding of the influencing mechanism, which is also more conducive to revealing how the joint action of psychological and physiological factors affects cognitive ability. In addition, considering that in current research, few studies specifically examine the obesity characteristics of large samples of young people, ignore how the interaction between psychology and physiology affects the consequences of obesity and do not pay attention to gender differences. In summary, this study aims to explore how BMI affects general cognitive ability of young adults by constructing a chain mediation model (Figure 1). The research hypotheses are as follows: H1: An increase in BMI will lead to a decrease in general cognitive ability in young adults; H2: Sleep quality mediates the relationship between BMI and general cognitive ability; H3: Impulsive trait mediate the relationship between BMI and general cognitive ability; H4: Sleep quality and impulsive trait will serve as chain mediators between BMI and general cognitive ability, i.e., high BMI reduces general cognitive ability by decreasing sleep quality and increasing impulsive trait; H5: Gender will moderate the impact of BMI on sleep quality and thus play a role in this chain mediation model.

## 2 Methods

### 2.1 Participants

This study used the HCP Release S1200 dataset. 1,206 Participants were recruited at Washington University in St. Louis between August 2012 and October 2015 (D. C. Van Essen et al.). This study was approved by the research ethics board of each institution and was conducted in accordance with the Declaration of Helsinki. All participants provided written informed consent and were young adults aged 22–35 years. The exclusion criteria were as follows: history of psychiatric disorder, substance abuse, neurodevelopmental disorder or damage, cardiovascular disease, severe health conditions (diabetes, multiple sclerosis, cerebral palsy, and premature birth), or magnetic resonance imaging contraindications (large tattoos, non-removable piercings, metal devices in the body, and claustrophobia). All participants were young adults with age between 22 and 35 years old. All participants provided written informed consent. All procedures were in accordance with the ethical standards of the responsible committee on human experimentation and with the Helsinki Declaration.

### 2.2 Clinical and neurocognitive assessments

In terms of clinical assessments, sleep quality was measured by the total score on the Pittsburgh Sleep Quality Index (PSQI), which assesses seven components of sleep: subjective sleep quality, sleep latency, sleep duration, habitual sleep efficiency, sleep disturbances, use of sleep medication, and daytime dysfunction. Higher scores of PSQI indicate lower levels of sleep quality (38).

Impulsive trait was assessed using the Delay Discounting Task (DDT), which measures the tendency to undervalue rewards that are delayed in time (39, 40). At the indifference points in the version of the DDT employed in the HCP, participants were equally likely to choose a larger reward later (e.g., $40 K in 3 years) or a smaller reward sooner (e.g., $200). The task utilized two initial reward amounts ($200 and $40 K) and six fixed delays (1, 6 months, 1, 3, 5, and 10 years) (41). The Area under the Curve (AUC) measure provided an index of how steeply an individual discounted delayed rewards. The AUC is the sum of the areas of 6 trapezoids; for each trapezoid, the area is calculated as (x2 − x1) ([y1 + y2]/2), where x1 and x2 represent the delays, and y1 and y2 are the subjective values associated with these delays. All x and y values were normalized by dividing by the largest x and y values, respectively, so the AUC ranged from 0 (maximum discounting) to 1 (no discounting), with a smaller AUC reflecting greater discounting and impulsivity (42). Given that individuals may have different perceptions of the value of $200 and $40 K due to their socioeconomic status, impulsivity in our study was measured as the average AUC for both the $200 and $40 K delayed discounting curves.

Neurocognitive ability was examined across several cognitive domains, including general cognitive ability and working memory. The fluid cognitive ability was derived by averaging the normalized scores of each of the Toolbox tests that are fluid ability measures, including Flanker, Dimensional Change Card Sort, Picture Sequence Memory, List Sorting and Pattern Comparison. Similarly, the crystallized cognitive ability was derived by averaging the normalized scores of each of the Toolbox tests that are crystallized measures, including Picture Vocabulary and Reading Tests. Hence, the general cognitive ability was derived by averaging the scores of the fluid cognitive ability and crystallized cognitive ability. Higher scores indicate higher levels of cognitive ability (43).

In our study, BMI was derived from self-reported height and weight data, as this was the method available in the dataset we utilized. The dataset has been previously validated and widely used in similar research contexts (44).

### 2.3 Statistical analysis

SPSS 24.0 was used to perform descriptive and correlation analyses of the study variables and demographic variables. Since the research variables in this study are continuous variables, Pearson correlation was used to explore the correlation between the variables. After that, PROCESS was used to test the mediating model and moderated chain mediation model (45). Parameter estimation was performed using the bootstrap method with 5,000 replicate samples and a confidence interval (CI) with a confidence level of 95%, which indicates that the corresponding effect is significant if the confidence interval (CI) does not include zero, and not significant if the confidence interval (CI) includes zero.

## 3 Results

### 3.1 Demographic characteristics, descriptive statistics and correlation analysis of variables

Demographic characteristics of the participants were shown in Table 1 and correlation analysis results for the variables were shown in Table 2. Impulsive trait was significantly negatively correlated with sleep quality and BMI, while being significantly positively correlated with general cognitive ability. Additionally, sleep quality showed significantly positive correlation with BMI and significantly negative correlation with general cognitive ability. Furthermore, BMI was significantly negatively correlated with general cognitive ability.

**Table 1:** Demographic characteristics of sample (*N* = 1,205).

Metric	M(SD) or percent
Age	28.83 (3.69)
Sex	
Female	54.36%
Male	45.64%
BMI	27.10 (5.88)
Impulsive trait	0.38 (0.23)
Sleep quality	4.85 (2.8)
General cognitive ability	112.20 (20.91)

M, mean; SD, standard deviation; BMI, body mass index; Sleep quality was measured by the total score on the Pittsburgh Sleep Quality Index; Impulsive trait was assessed using the Delay Discounting Task; General cognitive ability was derived by averaging the scores of the fluid cognitive ability and crystallized cognitive ability.

**Table 2:** Correlation analysis results of study variables.

	1	2	3	4	5
1.Impulsive trait	–				
2.Sleep quality	−0.18***	–			
3.BMI	−0.17***	0.12***	–		
4.General cognitive ability	0.22***	−0.13***	−0.19***	–	
5.Gender	0.03	−0.05	0.04	0.1***	–

***p < 0.001.

### 3.2 Mediation model testing

**Figure 2:**
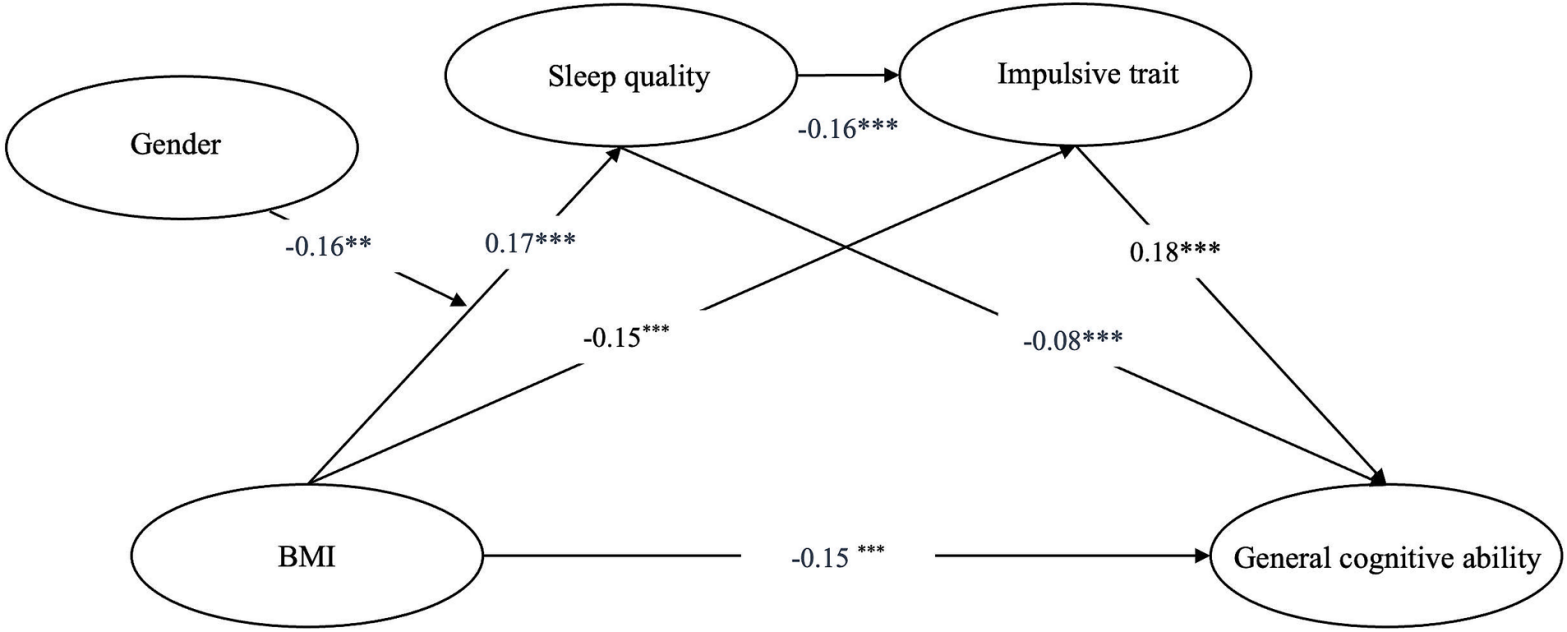
Moderated chain mediation model. BMI, body mass index. *p < 0.01, **p < 0.01, ***p < 0.001.

We used Model 6 from the SPSS macro (46) (Model 6 assumes that all paths in the chain mediation model are not moderated) to test the mediating roles of sleep quality and impulsive trait between BMI and general cognitive ability (see Figure 2; Table 3). The results showed that BMI was negatively correlated with general cognitive ability (*β* = −0.15, *p* < 0.001). BMI was positively correlated with sleep quality (*β* = 0.12, *p* < 0.001), and sleep quality negatively was negatively correlated with general cognitive ability (*β* = −0.08, *p* < 0.01). BMI was negatively correlated with impulsive trait (*β* = −0.15, *p* < 0.001), and impulsive trait was positively correlated with general cognitive ability (*β* = 0.18, *p* < 0.001). Additionally, sleep quality was negatively correlated with impulsive trait (*β* = −0.16, *p* < 0.001). The confidence intervals are not including 0, indicating that three pathway of mediating effects are significant. The results of the mediation effect analysis showed that the total indirect effect was −0.0389. The mediating effect of sleep quality was −0.0087, the mediating effect of impulsive traits was −0.0268, and the effect size of chain mediation was −0.034 (see Table 4).

**Table 3:** Tests for chained mediation effects.

Regression equation		Overall fit coefficient			Significance of coefficients		
Outcome variable	Predictor variable	*R*	*R* ^2^	F	*β*	95% CI	*t*
Sleep quality	BMI	0.12	0.01	15.82^***^	0.12	[0.06,0.17]	3.98^***^
Impulsive trait	BMI	0.23	0.05	33.66^***^	−0.15	[−0.20,-0.09]	−5.17^***^
	Sleep quality				−0.16	[−0.22,-0.11]	−5.73^***^
General cognitive ability	BMI	0.28	0.08	32.98^***^	−0.15	[−0.21,-0.09]	−5.25^***^
	Sleep quality				−0.08	[−0.13,-0.02]	−2.64^**^
	Impulsive trait				0.18	[0.13,0.24]	6.30***

BMI, body mass index. *p < 0.05; **p < 0.01; ***p < 0.001.

**Table 4:** Mediating effect and 95% confidence interval estimated.

Path		Indirect effect estimation	CI at 95% level
Total indirect effect		−0.0389	[−0.0557, −0.0242]
Indirect effect	BMI- SQ- GCA	−0.0087	[−0.0183, −0.0019]
	BMI-IT-GCA	−0.0268	[−0.410, −0.0145]
	BMI-SQ-IT-GCA	−0.034	[−0.0064, −0.0013]

BMI, Body mass index; SQ, Sleep quality; IT, Impulsive trait; GCA, General cognitive ability.

### 3.3 Moderation effect testing

**Figure 3:**
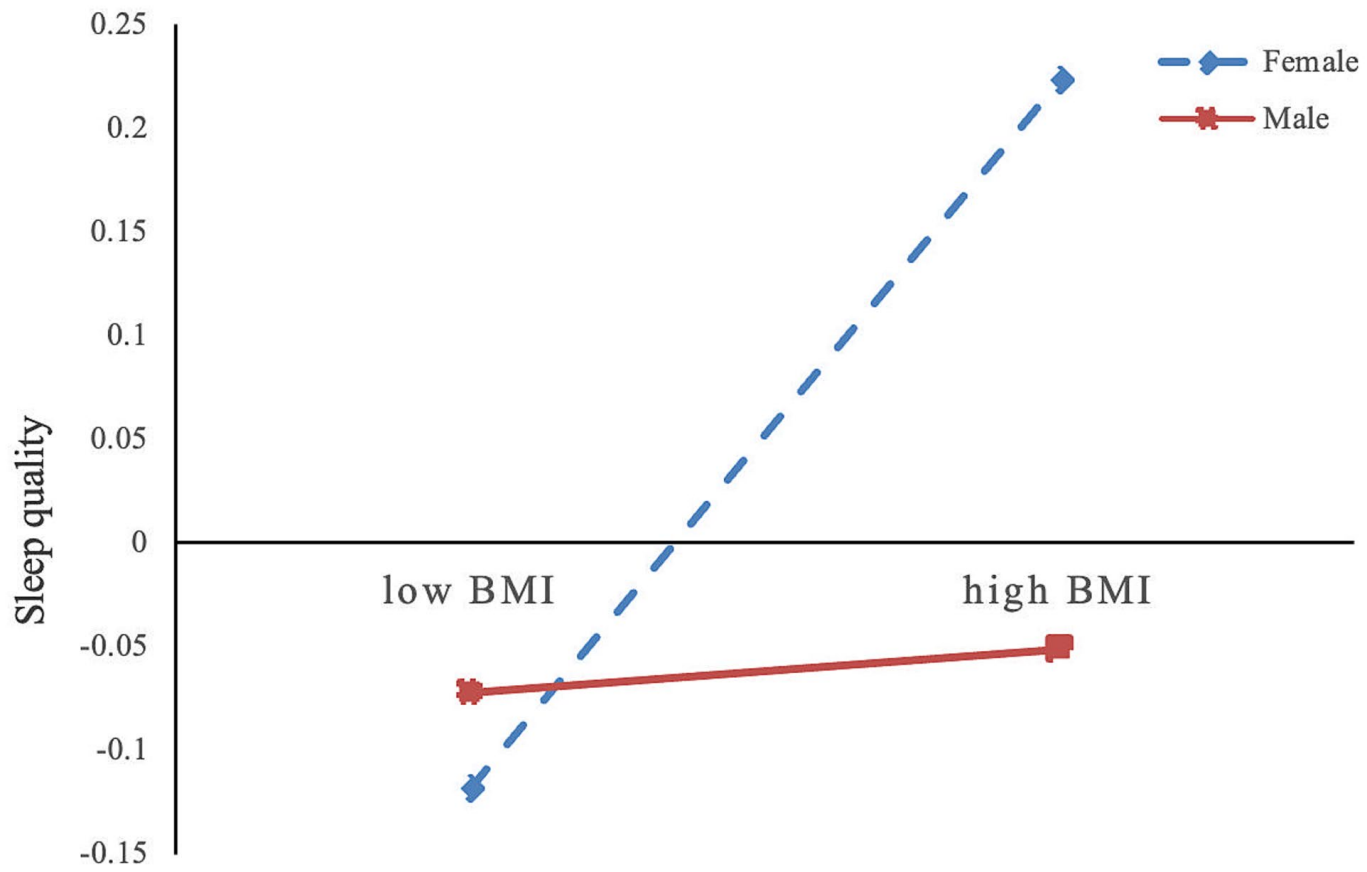
Moderating effects of gender on the relationship between BMI and sleep quality. BMI, body mass index.

We used Model 83 from the SPSS macro (46) (Model 83 assumes that all paths in the mediation model are moderated) to test the moderated mediation model (Figure 3). The results indicated that gender moderated the relationship between BMI and sleep quality (*β* = −0.16, *p* < 0.001), while the moderating effects on other paths in the mediation model were not significant. Further simple slope analysis showed that BMI’s effect on sleep quality was stronger in females (*bsimple* = 0.174, *p* < 0.001) compared to that in males (*bsimple* = 0.008, *p* = 0.872).

## 4 Discussion

This study found that higher BMI in young adults was associated with cognitive impairment. Further analysis revealed that sleep quality and impulsive trait acted as chain mediators between BMI and general cognitive ability. We also confirmed that gender is a moderating variable, with the effect of BMI on sleep quality being greater in females than in males, supporting our research hypotheses.

The results showed that BMI is negatively correlated with general cognitive ability in young adults, further supporting previous research that high BMI might be a significant risk factor for cognitive impairment in young individuals (47–49). Currently, previous studies have explained how BMI affects cognitive abilities from physiological perspectives, such as diabetes (9) and endocrine hormones (50). It also found that obesity in young individuals leads to iron deficiency, resulting in a decline in cognitive abilities (47–51). Additionally, due to young people’s pursuit of perfectionism but also due to their ongoing social comparison processes, where the combination of internal and external factors leads to catastrophic thinking, resulting in greater stress and anxiety (52, 53). Psychological stress and emotional problems can distract young students, making it difficult for them to concentrate on studies and tasks, resulting in a decline in cognitive performance (52). According to the “weight scarring hypothesis,” even after weight loss, past obesity is associated with poorer psychological outcomes, which may also lead to physical illness (54). This means that the process by which being overweight worsens an individual’s psychological state and impairs general cognitive ability may be long-lasting.

Second, the analysis showed that sleep quality mediated the relationship between BMI and general cognitive ability. High BMI is a risk factor for several sleep disorders and forms a vicious circle with poor sleep quality (37, 55). Sleep quality has been shown to influence cognition, particularly in children (14, 36, 56). Recent research on the relationship between BMI, sleep quality, and cognitive ability has predominantly focused on children (17). However, the underlying mechanism was still unclear in other populations. Our study is the first to reveal that the mediating effect of sleep quality in young adults, which offered us a new perspective and helped us to understand how BMI affects general cognitive ability in young adults.

We also found that impulsive trait mediated the relationship between BMI and general cognitive ability. High BMI often leads to increased impulsive trait, making it difficult for individuals to resist the need for immediate rewards (20). A review indicated that excessive food intake in individuals with high BMI is related to abnormalities in neuroendocrine function, which may be a trigger for impulsive behavior (21). In this study, impulsive trait were measured using a delay discounting task, involving brain regions such as the prefrontal cortex, striatum, and insula (25), which are related to the brain’s reward circuitry and mediate individuals’ high BMI and abnormal reward processing (57). That is, individuals with a high BMI are more likely to receive immediate rewards in the delay discounting task. In addition, high impulsive trait can lead to a decline in general cognitive ability. Impulsive trait are considered one of the four core factors impairing general cognitive ability, with attentional and non-planning impulsivity potentially lowering subjective general cognitive ability (24).

Our results also show that sleep quality and impulsive trait act as chain mediators between BMI and general cognitive ability. Specifically, an increase in BMI leads to lower sleep quality, which in turn increases impulsive trait, resulting in cognitive decline. Previous research in young populations has found that low sleep quality leads to increased impulsive traits (28, 31). Additionally, low sleep quality and high impulsive traits can jointly contribute to a decline in cognitive abilities (24). The results of this study further support these conclusions and provide an explanation of the relationship between BMI and cognitive ability.

Additionally, this study found that in the BMI-general cognitive ability pathway, gender moderates the impact of BMI on sleep quality, specifically showing that compared to men, women’s sleep quality is more likely to deteriorate with increasing BMI compared to men. The BMI-sleep quality-general cognitive ability pathway only holds true for women. One explanation for this is that the relationship between BMI and sleep quality in women often involves emotional eating behaviors, which are less evident in men. These behaviors make BMI and sleep quality interdependent in women (55). Second, hormonal differences between the sexes lead to differences in food intake behaviors and sleep–wake cycles, such as sleep duration, latency (time to fall asleep), and temporal structure. These differences make women’s sleep quality more vulnerable to increases in BMI (37, 58). Research has found that increased body fat is more directly detrimental to general cognitive ability (26), and women generally have higher body fat percentages than men, with weight gain contributing more to body fat increase (34, 59). Therefore, women’s general cognitive ability are more likely to be affected by BMI, which may be one of the factors why the BMI-sleep quality- general cognitive ability pathway is only valid for women. Additionally, previous research has found that men and women have different subjective reporting standards for sleep quality, with women more likely to report poor sleep quality (58). Therefore, analyses related to sleep quality may be influenced by subjective reporting differences. In summary, unlike previous studies on older adults or across all age groups (34, 37), this study specifically explored young adults and found that gender moderates the effect of BMI on sleep quality among this group, providing more evidence for understanding obesity in young populations.

Given the complex interactions between obesity and cognitive function, understanding these physiological and psychological mechanisms is crucial for developing effective prevention and intervention strategies. This study found that sleep quality and impulsive trait mediated the relationship between BMI and general cognitive ability, while gender moderated the relationship between BMI and sleep quality. We hope that the findings of this study can provide a scientific basis for future practical interventions to promote adolescent health. Given that our results show a strong association between cognitive decline and BMI and sleep quality, public health strategies should focus more on weight management and sleep health issues among young people (27), and provide activities or training that can help improve self-control and reduce impulsivity, such as mindfulness training (60, 61). Furthermore, it is important to consider gender differences and provide additional support for females, who are more vulnerable to the negative effects of increased BMI.

Limitations of this study include the following: First, the model is based on cross-sectional data, making it difficult to establish causal relationships between variables. Future longitudinal studies may provide stronger evidence. Second, there may be moderating variables other than gender in the model, which means that we may have missed other significant moderating effects. Future research can explore these additional variables. Third, sleep quality was measured using questionnaires, and as mentioned above, men and women have different subjective reporting standards for sleep quality. This discrepancy might cause the observed gender differences to deviate from objective reality. We hope that future research will use more objective methods of data collection. Fourth, our study only utilizes the HCP Release S1200 dataset. This chain mediation model can be validated across additional datasets to enhance its generalizability in the future. In addition, this study used BMI as a measure of overweight and obesity. Although BMI remains the most commonly used measurement standard worldwide and is widely recognized, it cannot distinguish between muscle mass and fat mass, nor does it reflect the specific distribution of body fat. Therefore, BMI may not be able to fully and accurately assess an individual’s obesity level and its impact on cognitive ability. Future studies should further consider body fat percentage or other more precise measurement indicators to more fully explore the relationship between obesity and cognitive ability.

## 5 Conclusion

BMI is significantly and negatively correlated with general cognitive ability, with sleep quality and impulsive acting as chain mediators between BMI and general cognitive ability. In addition, gender moderated the effect of BMI on sleep quality. The findings of this study not only provide a new theoretical perspective on the relationship between obesity and cognitive ability, but also provide practical guidance for health intervention, public health policy and individual health management. Future intervention strategies should not only focus on BMI management, but also combine sleep optimization and pay more attention to women’s sleep quality.

## Data Availability

The raw data supporting the conclusions of this article will be made available by the authors, without undue reservation.
